# P-wave dispersion: relationship to left ventricular function in sickle cell anaemia

**DOI:** 10.5830/CVJA-2010-047

**Published:** 2011-04

**Authors:** NI Oguanobi, BJC Onwubere, SO Ike, BC Anisiuba, EC Ejim, OG Ibegbulam

**Affiliations:** Department of Medicine, University of Nigeria Teaching Hospital, Enugu, Nigeria; Department of Medicine, University of Nigeria Teaching Hospital, Enugu, Nigeria; Department of Medicine, University of Nigeria Teaching Hospital, Enugu, Nigeria; Department of Medicine, University of Nigeria Teaching Hospital, Enugu, Nigeria; Department of Medicine, University of Nigeria Teaching Hospital, Enugu, Nigeria; Department of Haematology, University of Nigeria Teaching Hospital, Enugu, Nigeria

**Keywords:** P-wave dispersion, left ventricular function, sickle cell anaemia

## Abstract

**Background:**

The prognostic implications of P-wave dispersion in patients with a variety of cardiac disease conditions are increasingly being recognised. The relationship between P-wave dispersion and left ventricular function in sickle cell anaemia is unknown.

**Objective:**

This study was aimed at evaluating the relationship between P-wave dispersion and left ventricular function in adult Nigerian sickle cell anaemia patients.

**Methods:**

Between February and August 2007, a total of 62 sickle cell anaemia patients (aged 18–44 years; mean 28.27 ± 5.58) enrolled in the study. These were drawn from patients attending the adult sickle cell clinic of the University of Nigeria Teaching Hospital, Ituku-Ozalla, Enugu. An equal number of age- and gender-matched normal subjects served as controls. All the participants were evaluated with electrocardiography and echocardiography. P-wave dispersion was defined as the difference between the maximum and minimum P-wave duration measured in a 12-lead electrocardiogram.

**Results:**

P-wave duration and P-wave dispersion were significantly higher in patients than in controls. Significant correlation was demonstrated between P-wave dispersion and age in the patients (*r* = 0.387; *p* = 0.031). A comparison of subsets of sickle cell anaemia patients and controls with comparable haematocrit values (30–35%) showed significantly higher P-wave duration and P-wave dispersion in the patients than in the controls. The P-wave duration in patients and controls, respectively, was 111.10 ± 14.53 ms and 89.14 ± 16.45 ms (*t* = 3.141; *p* = 0.006). P-wave dispersion was 64.44 ± 15.86 ms in the patients and 36.43 ± 10.35 ms in the controls (*t* = 2.752; *p* = 0.013). Significant negative correlation was found between P-wave dispersion and left ventricular transmitral E/A ratio (*r* = –0.289; *p* = 0.023).

**Conclusion:**

These findings suggest that P-wave dispersion could be useful in the evaluation of sickle cell patients with left ventricular diastolic dysfunction. Further prospective studies are recommended to evaluate its prognostic implication on the long-term disease outcome in sickle cell disease patients.

## Summary

There is increasing recognition of the prognostic implications of the spatial variations of P-wave duration in normal individuals and patients with a variety of cardiac disease states.^1^-^3^ P-wave dispersion is defined as the deference between the maximum and minimum P-wave duration measured in a 12-lead electrocardiogram.^4^ It is related to the non-homogeneous and interrupted conduction of sinus impulse both intra- and interatrially and is considered a predictor of the occurrence of arrhythmias in patients with left atrial enlargement, left ventricular hypertrophy and left ventricular diastolic dysfunction,^5^ all of which are significant findings in sickle cell anaemia.^5^,^6^ There is scant information on this subject in the literature. The study is undertaken to evaluate the relationship between P-wave dispersion and left ventricular function in adult Nigerian sickle cell anaemia patients.

## Methods

A cross-sectional study was carried out on 62 sickle cell anaemia patients seen at the adult sickle cell clinic of the University of Nigeria Teaching Hospital (UNTH), Ituku-Ozalla, Enugu from February to August 2007. An equal number of age- and gender-matched normal subjects served as controls. All the participants were evaluated with electrocardiography and echocardiography.

Resting 12-lead electrocardiography was performed on all subjects using a Cardioline Ar-600 model electrocardiography machine at a paper speed of 25 mm/s and standardised at 0.1 mV/mm. A single observer analysed the electrocardiogram. The P-wave was measured from the beginning of the P-wave deflection from the isoelectric line to the end of the deflection returning to the isoelectric line. If the beginning or end of the deflection could not be satisfactorily defined, that lead was not used. The difference between the maximum and minimum P-wave duration was taken as the P-wave dispersion.^4^

Echocardiography was done using a Hewlett Packard Sonos 2500 echocardiography machine with 3.7-MHz transducer. The following measurements were taken in the standard positions as recommended by the American Society of Echocardiography:^7^,^8^ left atrial dimension, aortic root dimension, left ventricular end-systolic dimension, left ventricular end-diastolic dimension and end-diastolic volumes, velocities of E and A waves, isovolumic relaxation time and E-wave deceleration time, left ventricular ejection fraction, fractional shortening, and velocity of circumferential shortening.

Ethical clearance for the study was obtained from the ethics committee of UNTH, Enugu. Prior informed consent was obtained from all the participants in the study.

## Statistical analysis

Data were presented as means ± standard deviation. Comparison of continuous variables between the group of sickle cell disease patients and the control group was made with the independent Student’s *t*-test. In order to examine the effect of anaemia on the variables, the subjects were classified, based on the haematocrit values, into four classes in accordance with the World Health Organisation classification of anaemia as follows: class 1, normal (haematocrit ≥ 36%); class 2, mild anaemia (haematocrit 30–35.9%); class 3, moderate anaemia (haematocrit 21–29.9%); class 4, severe anaemia (haematocrit 18–20.9%).^9^

Inter-class differences in clinical, electrocardiographic and echocardiographic parameters in the patients were compared by one-way analysis of variance and *post hoc* multiple comparison of means using the Tukey’s honestly significant difference test. Intra-class differences in parameters between patients and controls in the same haematocrit class were analysed using the independent Student’s *t*-test. The relationship between P-wave dispersion and echocardiographic indices of left ventricular function (while controlling for the effect of anaemia) (haematocrit) was examined using the partial correlation analysis.

## Results

The mean ages of the patients and controls were 28.27 ± 5.58 (range 18–44) and 28.37 ± 5.91 (range 18–45) years, respectively. There were no statistically significant age and gender differences between patients and controls. The patients had statistically significant lower mean values than the controls in the measurement of height, body mass index and body surface area (*p* < 0.001, Table 1).

**Table 1.** Age, Gender And Anthropometric Data

*Parameters*	*SCA mean (SD)*	*Controls mean (SD)*	t*-test*	p*-value*
Age (years)	28.27 (5.58)	28.37 (5.91)	0.987	0.924
Gender (frequency (%))			0.00	1.00^a^
Male	31 (50)	31 (50)		
Female	31 (50)	31 (50)		
Total	62	62		
Weight (kg)	54.97 (10.61)	67.35 (8.37)	7.20	< 0.001*
Height (m)	1.62 (0.14)	1.72 (0.07)	4.960	< 0.001*
Body surface area (m^2^)	1.62 (0.03)	1.78 (0.14)	3.723	< 0.001*
Body mass index (kg/m^2^)	20.47 (2.73)	23.87 (3.22)	6.181	< 0.001*

*Statistically significant, ^a^Chi-square, SCA = sickle cell anaemia.

P-wave duration and P-wave dispersion were significantly higher in patients than controls (Table 2). Significant correlation was demonstrated between P-wave dispersion and age in the patients (*r* = 0.387; *p* = 0.031). When subsets of sickle cell anaemia patients and controls with comparable haematocrit values (30–35%) were compared, the patients were found to have significantly higher P-wave duration and dispersion than the controls. The P-wave duration in patients and controls, respectively, was 111.10 ± 14.53 ms and 89.14 ± 16.45 ms (*t* = 3.141; *p* = 0.006). P-wave dispersion was 64.44 ± 15.86 ms in the patients and 36.43 ± 10.35 ms in the controls (*t* = 2.752; *p* = 0.013).

**Table 2.** Comparison Of Electrocardiographic Characteristics Of Patients And Controls

*Variables*	*Values; mean (SD)*		t*-test*	p*-value*
	*SCA*	*Controls*		
Heart rate (beat/min)	80.61 (12.79)	68.98 (4.24)	6.327	< 0.001*
P-wave duration (ms)	128.0 (14.15)	90.30 (14.84)	14.189	< 0.001*
P-wave dispersion (ms)	65.7 (16.09)	34.7 (17.41)	9.014	< 0.001*

*Statistically significant, SCA = sickle cell anaemia.

In order to evaluate the effect of degree of anaemia on P-wave dispersion in the patients, the electrocardiographic parameters were compared among the haematocrit categories, as shown in Table 3. The haematocit values had no effect on the P-wave duration or dispersion.

**Table 3.** Electrocardiographic Parameters In Sickle Cell Anaemia; Effect Of Haematocrit Levels

*Parameters*	*Haematocrit levels; mean (SD)*			F*-statistic*	p*-value*
	*Mild*	*Moderate*	*Severe*		
Heart rate (beat/min)	83.56 (12.34)	77.65 (12.85)	83.27 (11.76)	1.391	0.297
P-wave duration (ms)	111.10 (14.50)	136.50 (17.31)	109.11 (16.40)	0.228	0.797
P-wave dispersion (ms)	64.42 (12.86)	64.04 (17.10)	60.02 (15.50)	0.245	0.784

*Statistically significant.

The result of a multivariate Pearson’s correlation analysis of P-wave dispersion and echocardiographic indices of left ventricular function are presented in Table 4. Of all the parameters evaluated, significant negative correlation was found between P-wave dispersion and left ventricular transmitral E/A ratio (*r* = 0.289; *p* = 0.023). The correlation was still significant after controlling for the effect of anaemia (*r* = 0.285; *p* = 0.027). Such correlation was not observed in the normal controls (*r* = 0.025; *p* = 0.859).

**Table 4.** Multivariate Pearson’s Correlation Of P-Wave Dispersion And Echocardiographic Parameters In Patients And Controls While Controlling For Haematocrit

*Variables*	*Patients*		*Controls*	
	*Pearson’s* r	p*-value*	*Pearson’s* r	p*-value*
LVMI	–0.0489	0.712	–0.0538	0.680
Fractional shortening	–0.1507	0.250	–0.1988	0.124
Ejection fraction	0.0489	0.711	0.0510	0.696
VCS	0.0607	0.645	–0.0541	0.679
E/A ratio	–0.285	0.027*	0.025	0.859
IVRT	0.1659	0.205	–0.0735	0.573
EDT	–0.01049	0.425	0.0005	0.997
Cardiac index	–0.2369	0.066	–0.0825	0.531

*Statistically significant; LVM = left ventricular mass, VCS = velocity of circumferential shortening, IVRT = isovolumic relaxation time, EDT = E-wave deceleration time.

## Discussion

This study revealed a significant increase in P-wave dispersion in sickle cell anaemia patients. The finding of a negative correlation between P-wave dispersion and left ventricular E/A ratio suggests that left ventricular diastolic function might be deranged in patients with increased P-wave dispersion. Reduced left ventricular relaxation and alteration in left ventricular chamber compliance are the haemodynamic abnormality for left ventricular diastolic dysfunction. Left ventricular hypertrophy, cardiomyopathy, myocardial ischaemia, systemic hypertension and normal aging are recognised causes. In diastolic dysfunction, rapid early filling is decreased, pressure upstream of the left ventricle is increased and atrial systole therefore has a crucial role to play to decrease atrial pressure and resume left ventricular filling.

P-wave dispersion has been described as a non-invasive indicator of risk of atrial fibrillation.^1^ If atrial fibrillation occurs, the loss of atrial systolic contribution to the left ventricular diastolic filling results in progressive diastolic dysfunction. Increased P-wave dispersion has been noted in hypertensive patients with diastolic dysfunction when compared with patients without diastolic dysfunction.^10^ Although some studies have suggested that left atrial diameter is an important predictor of atrial fibrillation, and that P-wave duration is related to left atrial dimension, the present study did not observe any relationship between P-wave dispersion and left atrial dimension.^11^,^12^

P-wave dispersion in sickle cell anaemia positively correlated with patients’ age (duration of illness), suggesting a progressive deterioration with time. Ayetemir and associates,^13^ in an investigation of clinical variables that affect P-wave dispersion in normal subjects, identified age as a related variable. However, in this study, no such correlation was observed in the control subjects. The authors are not aware of any previous study on P-wave dispersion in sickle cell anaemia. In view of the fact that diastolic dysfunction is a common cardiac complication of sickle cell anaemia, this study could be considered as an initial evaluation of the usefulness of this simple, non-invasive diagnostic tool in the assessment of diastolic function in sickle cell anaemia patients.

## Conclusion

P-wave dispersion was increased in patients with sickle cell anaemia and significantly correlated positively with age and negatively with left ventricular diastolic function. These findings suggest that P-wave dispersion could be useful in the evaluation of sickle cell patients with left ventricular diastolic dysfunction. However, further prospective studies are recommended to evaluate its prognostic implication on the long-term disease outcome in sickle cell disease patients.
